# Concordance and discordance of GeneXpert MTB/RIF and conventional culture method for diagnosis of Extra-Pulmonary Tuberculosis at a tertiary care hospital in Pakistan

**DOI:** 10.12669/pjms.40.2(ICON).8967

**Published:** 2024-01

**Authors:** Qurat-ul-Ain Zahid, Nazia Khursheed, Fareeha Adnan, Adeel Zafar

**Affiliations:** 1 Dr. Qurat-ul-Ain Zahid, Department of Pathology, Section of Microbiology Indus Hospital and Health Network, Karachi, Pakistan; 2 Dr. Nazia Khursheed, FCPS. Department of Pathology, Section of Microbiology Indus Hospital and Health Network, Karachi, Pakistan; 3 Dr. Fareeha Adnana, FCPS. Department of Pathology, Section of Microbiology Indus Hospital and Health Network, Karachi, Pakistan; 4 Dr. Adeel Zafar, Department of Pathology, Section of Microbiology Indus Hospital and Health Network, Karachi, Pakistan

**Keywords:** Mycobacterium tuberculosis, GeneXpert MTB/RIF, Extrapulmonary tuberculosis, Conventional culture, Pakistan

## Abstract

**Objective:**

To identify concordance and discordance between GeneXpert MTB/RIF assay and gold standard bacteriologic culture for the diagnosis of Mycobacterium tuberculosis (MTB) in Extra-Pulmonary tuberculosis (EPTB) specimens in our region.

**Methods:**

This is a retrospective cross-sectional study conducted at the Indus Hospital and Health Network. Data from 1^st^ January, 2020 to 31^st^ December, 2021 was analyzed. A total of 1499 EPTB specimens were included for which GeneXpert was requested along with acid-fast bacteria (AFB) culture from the same specimen. Specimens were processed according to specimen type following standard operating procedures of the laboratory. Fluorescent staining was performed on all specimens along with bacteriologic culture. The GeneXpert MTB/RIF assay was carried out in exact accordance with the manufacturer’s instructions.

**Results:**

Out of 1499 EPTB specimens, 1370 (91.39%) specimens exhibited concordance between GeneXpert and conventional culture method, while 129 (8.60%) specimens showed discordance. GeneXpert exhibited sensitivity and specificity of 69.4% and 94.3% respectively in comparison to culture.

**Conclusion:**

GeneXpert sensitivity for the diagnosis of EPTB varied with the site involved. Lower sensitivity was observed in ascitic and pleural fluids as compared to higher sensitivity observed among urine samples and pus aspirates. However, given the quick turnaround time and ease of use, it is a helpful tool in the diagnosis of EPTB when utilized in the appropriate clinical context. Caution is advised while interpreting negative GeneXpert results in endemic settings and should be interpreted along with other supporting clinical and diagnostic features.

## INTRODUCTION

According to the World Health Organization (WHO) global tuberculosis report of 2021 there were an anticipated 9.9 million diagnosed cases of tuberculosis, or an average 127 cases (114-140 per 100,000 people) reported worldwide in the year 2020. Regions with the highest rates of tuberculosis (TB) cases were South-East Asia (43%), Africa (25%), and Western Pacific region (18%). Eastern Mediterranean (8.3%), the Americas (3.0%), and Europe (2.3%) had the lowest reported cases. Eight of these 30 high TB burden countries accounted for two thirds of the total world population and accounted for 86% of all estimated incident cases across the globe: India (26%), China (8.5%), Indonesia (8.4%), the Philippines (6.0%), Pakistan (5.8%), Nigeria (4.6%), Bangladesh (3.6%), and South Africa (3.3%). Pakistan ranked 5^th^ amongst the 30 high TB burden countries and 4^th^ in highest prevalence of multidrug-resistant TB (MDR-TB) with an estimated 510,000 incident cases, which accounts for 61% of the TB burden in the Eastern Mediterranean Region.^1^,^2^ An upward trend is observed in extra-pulmonary tuberculosis (EPTB) cases in Pakistan, which accounts for 20% of all TB cases.^3^ Delayed or missed diagnosis, unsupervised, inappropriate and inadequate treatment regimens, poor follow-up and lack of a social welfare program are the key factors responsible for high burden and rising resistance.^1^

Mycobacterium tuberculosis (MTB) is one of the most ancient and deadliest bacteria, which remains a global threat despite the latest advancements in the area of diagnostics, treatment, and infection prevention and control.^4^-^7^ It causes tuberculosis (TB), a communicable disease which primarily affects the lungs but can disseminate to various organs such as gastrointestinal, genitourinary, cardiovascular and central nervous system, lymph nodes, joints, bones, and kidneys etc.^5^ Clinically TB is divided into Pulmonary tuberculosis (PTB) and Extra-Pulmonary tuberculosis (EPTB). Chief presenting complaints of PTB are chronic cough, and purulent blood-tinged sputum. EPTB a new emerging entity amongst children, and immunocompromised individuals, manifests as a chronic granulomatous disease affecting body tissues, and organs other than lungs. Patients with EPTB occasionally have a positive sputum culture but otherwise normal pulmonary function.^4^,^5^,^8^ Diagnosis of EPTB is challenging for both physicians, and pathologists, as the number of bacilli is often too low, and obtaining specimens from deep-seated infections can be difficult.^6^,^7^,^9^

Gold standard for definitive diagnosis of EPTB is culture on solid and liquid media. EPTB is a paucibacillary disease and culture requires only 10-100 bacilli/ml of concentrated sample. Yield of liquid culture medium is 10% more than solid media.^10^ The drawback of culture is prolonged turnaround time of result due to slow growth rate of MTB. It requires approximately six to eight weeks on LJ medium and, two to six weeks in liquid media.^5^,^11^,^12^ Moreover, it is a laborious process which requires specialized lab equipment of biological safety level III and its availability is limited in resource-constrained settings.^12^ GeneXpert MTB/RIF (Cepheid, Sunnyvale, CA, USA) is a fully automated real-time nested PCR assay, highly recommended by WHO for the diagnosis of PTB since 2010 and EPTB since 2013.^5^,^9^,^13^ It can detect both the MTB genome and rifampin resistance (rpoB gene mutation) in clinical specimens within two hours.^9^,^14^,^15^ Acid-fast bacteria (AFB) microscopy either with Ziehl-Neelsen stain (ZN) or fluorescent stain is time consuming, unable to differentiate MTB from Non-tuberculous Mycobacteria and, has been reported to have poor sensitivity and specificity due to paucibacillary nature of EPTB. Main objective of our study was to identify concordance and discordance between GeneXpert MTB/RIF assay and culture for the diagnosis of MTB in EPTB specimens. Our objective was to identify concordance and discordance between GeneXpert MTB/RIF assay and culture for the diagnosis of MTB in EPTB specimens.

## METHODS

This study was primarily laboratory based, conducted at the Indus Hospital and Health Network (IHHN), Karachi Pakistan. It was a retrospective, cross-sectional study, where data from 1^st^ January, 2020 to 31^st^ December, 2021 was extracted from Hospital Management Informatics System (HMIS) and Laboratory Information System. A total of 1499 EPTB specimens were included. The specimens included cold abscess (n=2, 0.13%), ascitic fluid (n=57, 3.80%), bone marrow (n=4, 0.26%), site not specified (n=30, 2%), cerebrospinal fluids (n=83, 5.53%), other fluids (n=75, 5%), gastric aspirate (n=84, 5.6%), pericardial fluids (n=2, 0.13%), peritoneal fluids (n=3, 0.2%), pleural fluids (n=302, 20.14%), pus aspirate (n=225, 15.01%), synovial fluids (n=2, 0.13%), granulation tissues (n=569, 37.95%) and urine (n=61, 4.06%). Specimens were screened by GeneXpert MTB/RIF, fluorescent staining and culture was performed on in-house prepared Lowenstein-Jensen (LJ) medium and commercially procured Mycobacterium growth indicator tube (MGIT), Becton Dickinson Biosciences, MD, USA. Standard operating procedures (SOPs) developed from WHO manual and manufacturers guidelines for MGIT and GeneXpert were strictly followed.^16^

### Ethics Committee approval

The study was conducted after taking approval from the ethics committee the Institutional Review Board Ref. (IRB) IHHN_IRB_2023_05_012.

### Inclusion criteria:

The data from all of the patients (inpatients and outpatients departments), where AFB Culture was requested along with GeneXpert MTB/RIF from the same Extra-Pulmonary Tuberculosis sample (EPTB), during the targeted time duration were included.

### Exclusion criteria:

- All Pulmonary-Tuberculosis (PTB) samples.
- All EPTB samples where GeneXpert was not requested with culture from the same sample.

### Statistical analysis

The data was entered and analyzed using the SPSS version 26.0 for windows. The normality distribution was assessed via Kolmogorov–Smirnov and Shapiro–Wilk tests. As per the distribution of data, the quantitative variables e.g., age was reported as mean ± standard deviation. Frequency and percentage were reported for categorical variables i.e., Gender, GeneXpert result and culture results. Sensitivity, specificity and predictive values were calculated by considering mycobacteriology culture as the gold standard, using 2x2 crosstab method on the SPSS software. Kappa analysis was also performed to see the agreement between GeneXpert and Culture results. P value <0.05 was considered statistically significant.

## RESULTS

**Fig.1:**
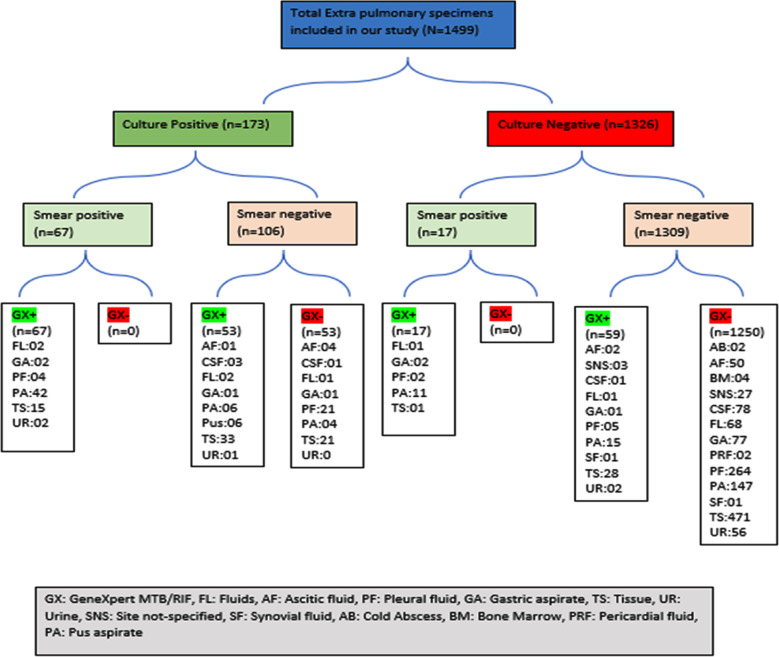
Distribution of specimens included in study according to culture positivity.

A total of 1499 EPTB specimens were analyzed. The minimum age of patients was one year while maximum was 87 years. The males were 46.8% while females were 53.2%. Mean age of patients in culture positive cases was 25.9±15 years with female predominance of 51.44%, while in culture negative cases it was 30±20 with a male predominance of 53.92%. (Table-I) Out of 1499 EPTB specimens’ positivity rates for microscopy, GeneXpert MTB/Rif, and culture were 5.6%(n=84), 13.07%(n=196), and 11.5%(n=173) respectively. Among the culture positive cases, 69.36% (n=120) were positive for GeneXpert, where pus samples comprised 45% (n=54), tissue 40% (n=48), pleural fluid 3.33% (n=4), other fluids 3.33% (n=4), CSF 2.5% (n=3), urine 2.5% (n=3), gastric aspirate 2.5% (n=3), and ascitic fluid 0.83% (n=1). Among culture positive cases, 30.63% (n=53) were negative for GeneXpert, where tissue samples and pleural fluid each comprised 39.62% (n=21), pus 7.54% (n=4), ascitic fluid 7.54% (n=4), CSF 1.88% (n=1), other fluids 1.88% (n=1), and gastric aspirate 1.88% (n=1). Among the culture-negative cases, 5.73% (n=76) were positive for GeneXpert MTB/RIF, where issue samples comprised 38.15%(n=29), pus 34.21% (n=26), pleural fluid 9.2% (n=7), gastric aspirate 3.94% (n=3), site not specified 3.94% (n=3), ascitic fluid 2.63% (n=2), other fluids 2.63% (n=2), urine 2.63% (n=2), synovial fluid 1.31% (n=1), and CSF 1.31% (n=1). Among culture negative cases GeneXpert was negative in 94.26% (n=1250) cases where tissue samples were 37.92% (n=474), pleural fluid 21.12% (n=264), pus 11.76% (n=147), CSF 6.24% (n=78), gastric aspirate 6.16% (n=77), fluids 5.44% (n=68), urine 4.48% (n=56), ascitic fluid 4% (n=50), site not specified 2.16% (n=27), bone marrow 0.32% (n=4), cold abscess 0.16% (n=2), pericardial fluid 0.16% (n=2), and synovial fluid 0.08% (n=1). The result of GeneXpert in culture positive and culture negative samples is shown in Fig.1.

**Table-I:** Demographic parameters of culture negative and culture positive cases.

Demographic parameters	Culture Positive cases (n=173)		Culture negative cases (n=1326)	
Age (Mean±SD)	25.9±15		30±20	
Gender	Male=83 (47.97%)	Female=89 (51.44%)	Male=715 (53.92%)	Female=605 (45.62%)

**Fig.2:**
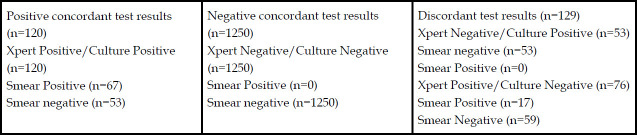
Concordant and Discordant test results between GeneXpert MTB/RIF and Culture.

The overall concordance of GeneXpert was seen in 91.39% (n=1370) and discordance in 8.26% (n=129) cases. In culture positive cases the concordance between GeneXpert and culture was seen in 69.36% (n=120) and discordance in 30.63% (n=53) specimens. In culture negative cases the concordance was seen in 94.26% (n=1250) and discordance was seen in 5.3% (n=76) (Fig.2). Kappa analysis was run to determine if there was agreement between GeneXpert and Culture. There was moderate agreement between GeneXpert and Culture κ = 0.602 p-value <0.01. Smear negative, Culture and GeneXpert positive were 50% (n=53) where tissue specimens represented 62.26% (n=33). The sensitivity and specificity of GeneXpert MTB/RIF in comparison to culture was 69.4% and 94.3% respectively with positive predictive value of 61.2% and negative predictive value of 95.9%. The GeneXpert was 98.7% specific and 75% sensitive in CSF. The sensitivity and specificity of GeneXpert in various samples is urine 100%, sensitive and 96.6% specific, pus 92.3% sensitivity and 85% specificity, fluids 80% sensitivity and 97.1% specificity, site not specified 90% specificity, gastric aspirate 75% sensitivity and 96.3% specificity, tissues 69.6% sensitivity and 94.2% specificity, and pleural fluid 32.3% sensitivity and 97.4% specificity. The sensitivity and specificity of GeneXpert in different samples is shown in Table-III.

**Table-II:** Diagnostic performance of GeneXpert in comparison to culture

	Culture	

	Positive	Negative
GeneXpert Positive	120	76
GeneXpert Negative	53	1250
Sensitivity	69.4%	
Specificity	94.3%	
PPV	61.2%	
NPV	95.9%	

**Table-III:** Diagnostic Performance of GeneXpert by specimen type.

Type of specimen	Number of Samples	GeneXpert			

		Sensitivity (%)	Specificity (%)	PPV (%)	NPV (%)
Abscess	2	0%	100%	-	100%
Ascitic fluid	57	20%	96.2%	33.3%	92.6%
Bone Marrow	4	0%	100%	-	100%
Site not-specified	30	0%	90%	-	100%
CSF	83	75%	98.7%	75%	98.7%
Fluids	75	80%	97.1%	66.7%	98.6%
Gastric aspirate	84	75%	96.3%	50%	98.7%
Pericardial fluid	2	0%	100%	-	100%
Peritoneal fluid	3	0%	100%	-	100%
Pleural fluid	302	32.3%	97.4%	58.8%	92.6%
Pus aspirate	225	92.3%	85%	64.9%	97.4%
Synovial fluid	2	0%	50%	-	100%
Tissues	569	69.6%	94.2%	62.3%	95.7%
Urine	61	100%	96.6%	60%	100%
Total	1499	69.4%	94.3%	61.2%	95.9%

## DISCUSSION

The overall sensitivity, specificity, positive and negative predictive value of GeneXpert results in our study were 69.4%, 94.3%,61.2% and 95.9%, respectively Table-II. The specificity of GeneXpert is consistent with studies conducted in other regions where specificity ranges from 73-100%.^5^ However, there is significant variation in sensitivity which ranges from 52-100%. The study conducted by Osei et al.^7^ in 2019 demonstrated 50%, and another study by Elbrolosy et al.^13^ in 2021 exhibited 81.6% sensitivity, while in our study it was 69.4% (Table-IV).^7^,^13^ This could be due to blood and other PCR inhibitory substances in samples with a paucibacillary disease and smashing of tissues during homogenization.^7^,^17^,^18^

**Table-IV:** Comparison of sensitivity and specificity of GeneXpert in EPTB specimens in different studies.

Study (year)	Country	GeneXpert sensitivity	GeneXpert specificity
Tortoli et al. (2012)^21^	Italy	81.3%	99.8%
Mechal at al. (2019)^9^	Morocco	78.8%	90.3%
Osei et al. (2019)^7^	South Africa	50%	97%
Elbrolosy et al. (2021)^13^	Egypt	81.6%	78.9%
Hillemann et al. (2011)^22^	Germany	77.3%	98.2%
Singh et al. (2016)^23^	India	88.4%	91.6%
Zeka et al. (2011)^14^	Turkey	64.6%	100%
Our study	Pakistan	69.4%	94.3%

The ranking of Pakistan among the top five high TB burden countries is alarming. A 20% increase in incident TB cases of EPTB further aggravates the situation. Directly observed therapy (DOT) strategy for TB which started in 2011 has been implemented in almost all the public health sectors. The National TB control program (NTP) revived under the Ministry of Health has developed uniform policies and strategies to counter the rising number of both PTB and EPTB cases in response to the declaration of TB as a national emergency in Pakistan.^17^,^19^ Despite all these measures, the diagnosis and management of EPTB remains challenging.

To the best of our knowledge, there is very little information on the performance of GeneXpert MTB/RIF assay in diagnosis of EPTB cases in Pakistan. This highlights the need of evaluating the diagnostic capability of GeneXpert in not only Urban Sindh but also in Rural Sindh and other provinces of Pakistan, as the number of EPTB cases may vary according to the burden of illness and its severity. The concordant results of GeneXpert have a great impact in early identification of TB in comparison with the culture which requires a prolonged time, but the number of discordant cases cannot be overlooked, addressing even a small number of cases is vital for lowering the disease burden and eradication of TB in endemic countries. Hence GeneXpert MTB/RIF cannot eliminate the necessity of conventional culture methods that are required to establish the diagnosis of TB.

Variation between sensitivities and specificities was observed according to the specimen type ranging from 20% sensitivity in ascitic fluids to 92.3% in pus aspirates and, 50% specificity in synovial fluids to 100% in cold abscess, bone marrow, pericardial and peritoneal fluids. Our results showed higher pus sensitivity of 92.3% compared to 56.7% demonstrated in a study by Parkash et al.^15^ when compared to a study by Vadwai et al.^8^ where CSF material showed a subpar sensitivity of 29%, the CSF specimens in our study demonstrated superior sensitivity of 75%.^8^ In a study conducted in Lahore, Pakistan by Iram S et al.^12^ the sensitivity and specificity of GeneXpert in EPTB specimens was reported as 100% and 86%, respectively.^12^ This is in contrast with our results where sensitivity was 69.4% and specificity was 94.3%. The stark difference between both studies can be attributed to the difference of sample size, with our sample size being significantly higher than the study in comparison. In another study conducted in Peshawar, Pakistan by Khan AS et al.^20^ the overall sensitivity of GeneXpert was 73% and specificity was 100%. In terms of specimens, the tissue samples in our investigation showed a sensitivity of 69.6% compared to 100% by Khan AS et al.^20^ 75% compared to 83% for CSF samples, and 100% compared to 57% for urine samples. The heterogeneity between sensitivities in different studies can be attributed to the difference in the disease burden, patient populations, type of EPTB, sample size and specimen quality, in countries where TB is endemic.

### Limitations

It is a single center study thus its findings cannot be generalized as the data may vary. Secondly, this was a retrospective, cross-sectional study, hence the possibility of handling error, processing of specimens, observer’s bias in microscopy, and technical errors related to GeneXpert could not be repeated for confirmation. For samples labelled as site-not-specified and other fluids, the nature and anatomic location of samples could not be verified. Furthermore, radiologic evidence and histopathological findings could not be compared and sensitivity and specificity of GeneXpert MTB/RIF assay for rifampin resistance was not evaluated.

## CONCLUSION

The performance of GeneXpert varied with the site of extrapulmonary involvement with lower sensitivity in ascitic and pleural fluids and a higher sensitivity in pus aspirates, cold abscesses and urine specimens. The rapid turnaround time of GeneXpert can help in timely detection of tuberculosis in these cases and appropriate therapeutic intervention can be started before culture results are available. However, in areas where TB is endemic, caution is advised for interpreting negative GeneXpert results in clinical settings and should be interpreted along with clinical signs and symptoms, positive contact history, radiological findings, histopathological diagnosis and microbiological culture. Further research with larger sample size is needed to evaluate the utility of GeneXpert in EPTB cases in endemic settings, which will aid in development of concrete diagnostic guidelines for effective treatment.

### Authors` Contribution:

**QZ, & NK** Involved in conception, design, , data collection and manuscript writing. **AZ** did statistical analysis & editing of manuscript. **NK and FA** Review, editing and final approval of manuscript. All authors contributed in critically revising the manuscript and are accountable for the accuracy or integrity of the research.
